# CLSQL: Improved Q-Learning Algorithm Based on Continuous Local Search Policy for Mobile Robot Path Planning

**DOI:** 10.3390/s22155910

**Published:** 2022-08-08

**Authors:** Tian Ma, Jiahao Lyu, Jiayi Yang, Runtao Xi, Yuancheng Li, Jinpeng An, Chao Li

**Affiliations:** College of Computer Science and Technology, Xi’an University of Science and Technology, Xi’an 710054, China

**Keywords:** Q-learning, mobile robot, path planning, complex environment, prior knowledge

## Abstract

How to generate the path planning of mobile robots quickly is a problem in the field of robotics. The Q-learning(QL) algorithm has recently become increasingly used in the field of mobile robot path planning. However, its selection policy is blind in most cases in the early search process, which slows down the convergence of optimal solutions, especially in a complex environment. Therefore, in this paper, we propose a continuous local search Q-Learning (CLSQL) algorithm to solve these problems and ensure the quality of the planned path. First, the global environment is gradually divided into independent local environments. Then, the intermediate points are searched in each local environment with prior knowledge. After that, the search between each intermediate point is realized to reach the destination point. At last, by comparing other RL-based algorithms, the proposed method improves the convergence speed and computation time while ensuring the optimal path.

## 1. Introduction

Currently, with the development of Industry 4.0, there is an increasing demand for intelligent equipment upgrades in various industries. Mobile robots, as an integral part of the intelligent industry, have been applied to serve indispensable roles in different fields [1]. Path planning is the central crux in the research field of mobile robots such that they know how to move from source to target and how to execute the desired task. The mobile robots not only need to complete tasks but also have to be able to avoid various obstacles, which will adversely affect their performance. Therefore, path planning is important for mobile robots.

Since path planning problems were proposed, many scholars have proposed many representative algorithms, such as Dijkstra [2], A* [3], Rapidly-exploring Random Trees (RRT) [4], Genetic Algorithm (GA) [5] and Particle Swarm Optimization (PSO) [6], etc. In addition, there are also popular methods based on optimal control theory, such as coevolutionary multipopulation genetic algorithm (CMGA) [7], CMGA takes a time-optimal path planning approach to complete the cooperation between vehicles, and Xiaoshan Bai [8] utilized the accessible area analysis and optimal control theory to generated the time-optimal path. With the deepening of research and technological progress, the speed and accuracy of path planning have been enhanced. However, in the complex environments, the above algorithms have shortcomings such as low planning efficiency and easily fall into locally optimal solutions [9]. Therefore, in order to overcome these shortcomings, machine learning techniques endow new solutions in the field of path planning.

With the development of artificial intelligence, many scholars used machine learning (ML) methods to solve the above problem. ML receives a large amount of information data [10], establishes connections based on data analysis, and finally obtains predictions. Reinforcement Learning (RL), as a kind of ML method for interacting with the environment [11], was initially applied in game theory, information theory, and control theory. Through time, it has been used in mobile robots. When mobile robots interact with the environment, they deliver feedback in the form of the reward information according to the environment, select the optimal action, and finally obtain the path planning result. RL can be divided into on-policy and off-policy according to the way of policy learning. In the on-policy, the behavior policy is identical to the target policy. In the off-policy, in contrast, they are different. Such as Q-Learning algorithm, the off-policy means that the behavior policy is ε-greedy and the target policy is greedy. On-policy mainly includes Policy Gradient [12], SARSA [13], Actor-Critic [14] algorithms, and off-policy includes Q-Learning [15] and DQN [16] algorithms, among which the Q-Learning algorithm is widely used in various robotics fields.

The Q-Learning (QL) algorithm adopts a time series differential method of off-policy [17]. Off-policy means that agents explore diversified data through behavior policy in the process of interacting with the environment, so as to continuously optimize the target policy and finally obtain a global optimal value. The sequential difference method approximates the present state-action value function maxQ(s,a) by learning the state-action value function Q(s,a) of the subsequent state to realize the search of the unknown environment. Mobile robots observe and adjust their actions by observing the environment. However, the major limitations are the amount of time and convergence speed required in complex environments.

Ensuring that the mobile robot completes path planning within a limited time is the main problem that QL needs to face at present. Therefore, in this paper, we propose an improved Q-learning algorithm called CLSQL. The main contributions of this paper are as follows:

1 We introduce the concept of the local environment and establish the improved Q-learning based on a continuous local search policy. Specifically, the local environment and intermediate points are gradually determined in the search process to improve the search efficiency and reduce the number of iterations.
2 Based on the local search policy, the proposed method adds a prior knowledge and optimizes a dynamically adjusting ε-greedy policy.
3 We prove that compared with other RL algorithms, the proposed algorithm can not only ensure the search efficiency and the iteration number but also achieve satisfying results under different sizes and complexity of the map. The multigroup simulation results verify our analysis.

The remaining chapters of this paper are as follows: In Section 2, we introduce the related work of path planning in RL. In Section 3, we describe the method of environment modeling. In Section 4, we mainly introduce the motivation, the specific operation of the proposed algorithm. In Section 5, we discuss the three kinds of experiments conducted with different purposes and analyze and discuss the results, and in Section 6, we summarize the work of this paper and discuss some future directions derived from it.

## 2. Related Work

Regarding path planning problems, many scholars have verified and recognized the RL algorithms, but they also have limitations. First, when the size of the environment increases, not only is a larger adaptive memory matrix required and more computation time is needed to update the Q-value matrix but also the convergence speed will decline sharply. Second, in the early stages of the search, the algorithms will perform blind and invalid attempts and searches. If the worst case happens, all the state–action pairs in the environment may be searched. Therefore, how to increase the search efficiency and convergence speed of the QL algorithm in path planning is a common challenge for scholars [18,19,20].

### 2.1. The QL-Based Path Planning Method

The core idea of the QL algorithm is that when updating the Q value of a state–action pair, it adopts the Q value of the next state–action pair generated by the policy to be evaluated rather than the Q value of the next state–action pair that follows the present policy. Specifically, in the path planning problem, the mobile robot carries out random sampling in the environment and generates paths through multiple sampling. During this period, the behavior policy and target policy interaction iterate until the optimal path is obtained. Algorithm 1 describes the learning process of the QL algorithm. The Q-table update process by (1) is as follows:

(1) $$Q\left(s,a\right)\leftarrow Q\left(s,a\right)+\alpha \left[r+\gamma \max_{a^{\prime }}Q\left(s^{\prime },a^{\prime }\right)-Q\left(s,a\right)\right],$$

where *s* is state, *a* is action, *r* is the reward that received a reinforcement signal after *s* is executed, $s^{\prime }$ is next state, γ(0≤γ<1) is discount factor, and α(0≤α<1) is learning rate.
**Algorithm 1: The Q-learning Algorithm**1   Initialize $\underset{\;n\times \;m\;}{Q}\left(s,a\right)=0$ (n states and m actions)2   Repeat3     Using ε-greedy to select *a* from present state *s*;4     Take action *a*, get *r*, $s^{\prime }$;5     Update Q(s,a) by (1)6     $s\leftarrow s^{\prime }$7   Until *s* is destination

The solution to the above problems has seen numerous methods. For example, Bo Zhang [21] used the QL algorithm to dynamically adjust the robot’s local path, optimize the local real-time detection ability of the robot, and avoid the problem that the robot is close to obstacles. The Deep Q-Network (DQN) [22] takes the state–action pair as the input of the neural network, obtains the Q value of the action after neural network analysis, and replaces the Q-table with a neural network to reduce the information dimension. Ee Soong Low [23] proposed the Flower Pollination Algorithm (FPA) to improve the initialization of the Q-table. Chen Chen [24] predicted the position and direction of ships in a future period of time through the first-order Nomoto model to establish a Q-table of relevant ships. Meng Zhao [25] combined empirical memory of the Q-table to guide agents to search in unknown environments through dual cues. Juli Zhang [26] divided the global environment into four local environments and initialized the Q-table by artificially judging the destination to accelerate the search process. Abderraouf Maoudj [27] optimized the initialization mode of the Q-table. Meanwhile, to avoid useless search, he innovated an efficient selection policy by rectangling the obstacle and adding a safe distance. Reasonable actions were selected by judging the vertex position of the obstacle on the predicted path. Pradipta Kumar Das [28] used the delayed update to save considerable space for the Q-table, thus reducing the turning angle of the path.

### 2.2. The RL-Based Path Planning Method

Moreover, other RL algorithms and their optimizations also have a great amount of research in path planning. The SARSA(λ) [29] algorithm is an on-policy algorithm based on the qualification trace. It learns experience by learning and updating each step that has occurred before; that is, it will update the previous step at the same time as updating the present step. The advantage of this algorithm is that it can learn the optimal policy more quickly and effectively through different degrees of learning for all steps. The strength of the Actor–Critic (AC) algorithm is to select the appropriate action in the continuous action. Can Xu [30] adopts the AC algorithm in the trajectory planning of autonomous vehicles, which makes trajectory planning more flexible, safe, and efficient. The Deep Deterministic Policy Gradient (DDPG) algorithm is based on the AC; Joohyun Woo [31] used DDPG to capture the experience of the unmanned surface vehicle in the path tracking test. Although the above literature has different solutions to the path optimization problem of mobile robots, most of them do not involve how to solve the problem in a complex environment, so quickly obtaining the optimal path in a complex environment is still the most necessary research at present.

In addition, for algorithms like RL, learning is an iterative process. Not only the time spent in a single successful iteration but also the total time spent on the entire iteration process should be considered. To date, there is still no good method to obtain the optimal path with fewer iterations. The number of iterations of a learning process increases exponentially, especially when the environment of mobile robots becomes increasingly complex [32]. Therefore, in this paper, we propose a continuous local search policy based on the QL algorithm (CLSQL), because the QL algorithm has low search efficiency in a complex environment but high search efficiency in a simple environment.

## 3. Environment Modeling Based on Grid Map

In the section, we adopt the grid method [33] to model the working environment of mobile robots. The grid method is characterized by a simple data structure and an effective expression of spatial variability. Then, we formulate the path planning problem to better display the path planning results in a two-dimensional (2D) environment.

### 3.1. Grid Method for 2D Environment Modeling

The grid method is the most commonly used environment modeling method for path planning of mobile robots, which can simply and accurately represent various information on maps. In this paper, the grid method is used to establish a 2D environmental coordinate system, including a starting coordinate, a destination coordinate, free coordinates, intermediate coordinates, and obstacle coordinates.

In a n×n rectangular environment, *n* is the number of rows and columns in the environment, including a total of $n^{2}$ grids. *R* is used to represent the cost of the present grid, and $R_{ij}$ represents the reward of the grid in row *i* and column *j*. The reward function of the specific grid is shown in (2):

(2) $$R_{ij}=\left\{\begin{array}{cc} -5, & r_{\mathrm{start},\;}\\ 50, & r_{\mathrm{end},\;}\\ 0, & r_{\mathrm{road},\;}\\ 30, & r_{\mathrm{mid},\;}\\ -50, & r_{\mathrm{obs}.\;} \end{array}\right.$$

*G* is used to represent all reward matrix in the 2D rectangular environment, and the numerical matrix of the cost in the environment is shown in (3):

(3) $$G=\left[\begin{array}{ccc} R_{11} & \ldots & R_{1n}\\ \cdots & \cdots & \cdots \\ R_{n1} & \ldots & R_{nn} \end{array}\right].$$

**Figure 1:**
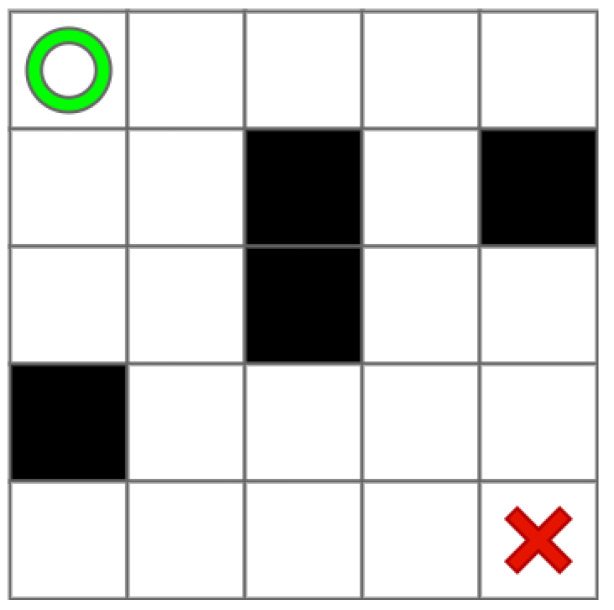
An example of 2D environment by grid map.

For example, in the 2D environment map shown in Figure 1, the green circle is the starting point, the red cross is the destination point, and the black grid is the obstacle.

### 3.2. Problem Formulation

Here, we describe the proposed algorithm settings. The mobile robot should move from its starting position $s_{start}\left(x_{s},y_{s}\right)$ to its destination position $s_{E}\left(x_{E},y_{E}\right)$. The aim is to find a planned path connecting $s_{start}$ to $s_{E}$ that avoids colliding with any static obstacle points $\left(s_{Obs}^{j}\left(x,y\right),j=1,\ldots ,m\right)$, where the planned path consists of a set of *n* continuous points $\left(P_{i}\left(x_{p},y_{p}\right),i=1,\ldots ,n\right)$ and $s_{Obs}$ consists of a set of *m* discrete points. During the search process, the algorithm will gradually determine the intermediate points $s_{I}\left(x,y\right)$, where $s_{I}$ consists of a set of *k* points.

The following notations describe indices and parameters used in the mathematical model:

• *n*: number of points of the planned path,
• *m*: number of $s_{Obs}$ in the global environment,
• *k*: number of $s_{I}$,
• $P_{i}\left(x_{p},y_{p}\right)$: $i^{th}\left(i\in \left\{1,\ldots ,n\right\}\right)$ point of the planned path.

The decision variables of the mathematical model are as follows:

(4) $$\mathrm{Minimize}\left(\sum_{i=1}^{i=n-1}\sqrt{{\left(x_{p}^{i+1}-x_{p}^{i}\right)}^{2}+{\left(y_{p}^{i+1}-y_{p}^{i}\right)}^{2}}\right),\forall i\in \left\{1,\ldots ,n-1\right\},$$

Subject to:

(5) $$\left(x_{p}^{i+1}\neq x_{p}^{i}\right)\vee \left(y_{p}^{i+1}\neq y_{p}^{i}\right),\forall i\in \left\{1,\ldots ,n-1\right\},$$

(6) $$P_{i}\left(x_{p},y_{p}\right)\neq s_{\mathrm{Obs}}^{j}\left(x,y\right),\forall i\in \left\{1,\ldots ,n-1\right\},\forall j\in \left\{1,\ldots ,m\right\}.$$

The objective function, given by (4), consists of minimizing the length of the planned path. The constraint given by (5) requires all points to be different. Constraints in (6) ensure that the planned path points do not overlap environment obstacles. It should be noted that $\left(x_{p}^{i},y_{p}^{i}\right)$ is the same as $P_{i}\left(x_{p},y_{p}\right)$, but it is convenient to show in (4).

## 4. The Continuous Local Search Q-Learning Algorithm

**Figure 2:**
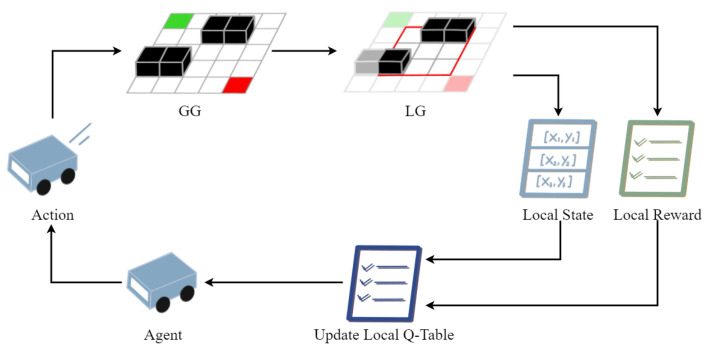
Learning policy of the CLSQL.

To solve the problems of slow convergence speed and a large number of iterations in the QL algorithm, in this paper, we propose an improved Q-learning algorithm based on a continuous local search policy, which is named the CLSQL. The CLSQL algorithm is mainly improved in three ways: (1) A prior knowledge is established through Euclidean functions to change the initialization of the Q-table. (2) The local search policy is adopted to improve the number of iterations of the QL algorithm. (3) Enhance the efficiency of each search by a modified dynamically adjusting ε-greedy policy. Figure 2 shows the method of interaction between the agent and the environment in the CLSQL algorithm.

### 4.1. Motivation

In contrast to most algorithms that need to search for the entire environment, CLSQL can be searched at the local environment, and then the previous search path is connected together. When the mobile robot completes the search in a local environment, the subsequent search does not affect the completed planned path. It reduces invalid search and increases convergence speed.

### 4.2. Environmental Prior Knowledge Based on Euclidean Distance

There is no prior knowledge of the environment in the QL algorithm, and all Q-tables in the initial state are equal to 0. Therefore, in the early stages of the search, the action selected in each step of the policy is tentative. Meanwhile, the sparse nature of the reward function leads to slow convergence speed and many iterations. In path planning problems, rewards are updated only when the destination is reached or an obstacle is encountered. Especially in the face of a huge and complex environment, there is a large amount of negative or invalid search space. Therefore, effective Q-table updates have become increasingly important.

In this paper, the distance function is used to determine the prior knowledge of the environment. Because the action group adopted by the proposed algorithm includes eight directions: up, down, left, right, top left, top right, bottom right, and bottom left, the European distance is more suitable for the actual map environment. Algorithm 2 shows the process of obtaining prior knowledge. The 2D Euclidean distance is a method to calculate the sum of the absolute wheelbase of two points $p_{1}\left(x_{1},y_{1}\right)$ and $p_{2}\left(x_{2},y_{2}\right)$ in the standard coordinate system, as shown in (7):

(7) $$D=\sqrt{{\left(x_{2}-x_{1}\right)}^{2}+{\left(y_{2}-y_{1}\right)}^{2}}.$$

**Algorithm 2: Prior Knowledge**1   Initialize Agent State s(x,y), Destination State $s_{E}\left(x_{E},y_{E}\right)$, Obstacle State $s_{Obs}$2   Repeat3     if *s* == $s_{Obs}$ then4        $D_{s}=0$5     else6        $D_{s}=\sqrt{{\left(x_{E}-x\right)}^{2}+{\left(y_{E}-y\right)}^{2}}$7   Until All *s* traversal completed

**Figure 3:**
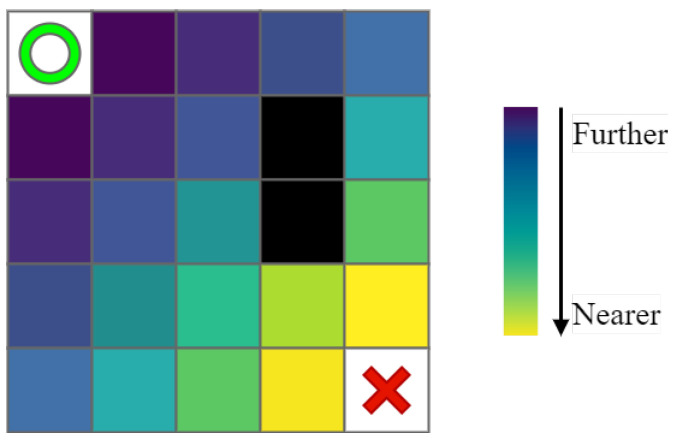
Prior knowledge based on Euclidean distance.

The improved algorithm obtains prior knowledge and initializes the Q-table through the following steps. The details are as follows:

1 First, determine the starting and destination points in the environment and calculate the distance between all points except obstacles and the starting point through the Euclidean function, such as shown in Figure 3, where the green circle, the red cross, and the black squares represent the starting point and destination point and obstacles, and the rest of the color squares from deep to shallow represent the size of the prior knowledge of the present points: the deeper the color, the further away from the destination point.
2 Then, with the agent’s continuous search, the new state–action value is added to the Q-value of the relevant state, and the prior knowledge $D_{s}$ relevant to the present state is added, which is defined as:
  (8) $$Q\left(s,a\right)=\left\{\begin{array}{c} \frac{1}{D_{s}},\;D_{s}>0,\\ 0,\;D_{s}=0. \end{array}\right.$$
3 Finally, the Q-table with prior knowledge is used to learn and select the optimal action policy in the present environment.

### 4.3. Efficient Local Search Policy

As the size of the environment increases, the dimensions involved in the QL algorithm also increase. Although the RL algorithm can reach the destination point through continuous iteration and search in the environment, efficient path planning cannot be carried out. At the same time, the repeated search of a large amount of invalid information in the environment will also affect the iterative efficiency of the algorithm. Therefore, the local search policy proposed in this paper can simplify the complex environment by identifying $s_{I}\left(x,y\right)$ and gradually searching in the simple local environment, thus reducing a large number of invalid search spaces and increasing the iterative efficiency of the proposed algorithm. The specific operations are as follows:

1 By setting a Local Environment Size(LS) size based on the start point $s_{start}\left(x,y\right)$ or $s_{I}\left(x,y\right)$ in the Global Environment Grid(GG).
2 Based on the centering of the point $s_{start}\left(x,y\right)$ or $s_{I}\left(x,y\right)$, a 2D matrix was established and diffused to the size of LS, finally obtaining the Local Environment Grid(LG). Particularly, the first LG is determined by $s_{start}\left(x,y\right)$ and then by $s_{I}\left(x,y\right)$.
3 In the present local environment, the $s_{I}\left(x,y\right)$ are determined based on prior knowledge.
4 As the search progresses and point $s_{I}\left(x,y\right)$ is updated, the complex environment can be transformed into several continuous local environments.

The local environment was determined by Algorithm 3, to limit the algorithm search space in the search space effectively and reduce the invalid search space. Then, the search effectiveness and purpose of the algorithm were increased by determining $s_{I}$ in the local environment, thus increasing the iteration speed of the algorithm. Equation (9) describes how to use the Priority Queue (PriQ) to determine the priority of the $s_{I}$. Algorithm 4 describes the method of determining $s_{I}\left(x,y\right)$ for the proposed algorithm.

(9) $$\mathrm{PriQ}=D_{s}+\frac{GS}{\left|r_{obs}\right|}P_{s},$$

where GS is the Global Environment Size, $P_{s}$ is the sum of reward in eight neighborhoods of the state *s*, and the coefficient of the second term is employed to keep the order of magnitude of cost the same as $D_{s}$.
**Algorithm 3: Local Environment**1   Initialize LG, LS, $s_{start}\left(x,y\right)$, $s_{I}\left(x,y\right)$2   for i=$\left\lceil -\frac{LS}{2}\right\rceil ,\ldots ,0,\ldots ,\left\lfloor \frac{LS}{2}\right\rfloor$ do3     for j=$\left\lceil -\frac{LS}{2}\right\rceil ,\ldots ,0,\ldots ,\left\lfloor \frac{LS}{2}\right\rfloor$ do4        if i+x≥0&i+x<GS&j+y≥0&j+y<GS then5          $LG_{i+x,j+y}\leftarrow GG_{i+x,j+y}$6     endfor7   endfor


**Algorithm 4: Intermediate Points**
1   Initialize $s_{E},s_{I}$2   if $s_{E}\in LG$ then3     $s_{I}=s_{E}$4   else5     using (9) to sort the priority of the local environment points;6      $s_{I}$ is the relevant point to the minimum value in PriQ.7   Return $s_{I}$

**Figure 4:**
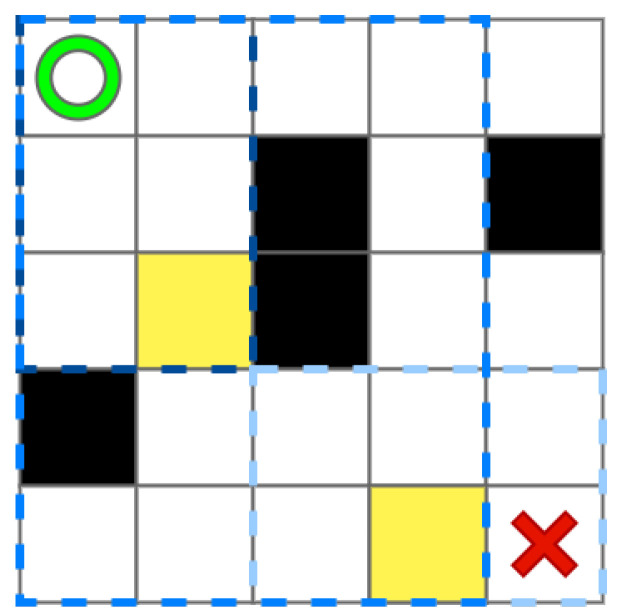
The grid map with intermediate point.

Based on Figure 1, we use Figure 4 show the process of getting $s_{I}$. Here, the LS = 5, and there are three different blue local environments that the size is 2×3, 4×5, and 3×2, respectively. Due to the limits of the small map boundaries, the full effect cannot be achieved. $s_{I}$ is represented by the yellow grid.

### 4.4. Search and Iteration Process of the CLSQL Algorithm

On the premise of prior knowledge and the local environment, the CLSQL algorithm will adopt a method similar to the ε-q-learning [34] algorithm to conduct spatial search and policy learning, but the difference is that the CLSQL algorithm learns in a continuous local environment. The ε-q-learning algorithm optimizes the search process by dynamically adjusting ε-greedy policy. Different from the ε-q-learning, in this paper, we modified the dynamically adjusting ε-greedy policy, that is, (i) the greedy factor is initialized at the beginning of each local environment search; then, (ii) the greedy factor is increased when the agent collides with obstacles, or (iii) the greedy factor is decreased when the search result in the local environment is the nonoptimal path. In this paper, according to experience, the ε-greedy is initialized to 0.9 every time, and the extent of increase or decrease is 0.01.

**Figure 5:**
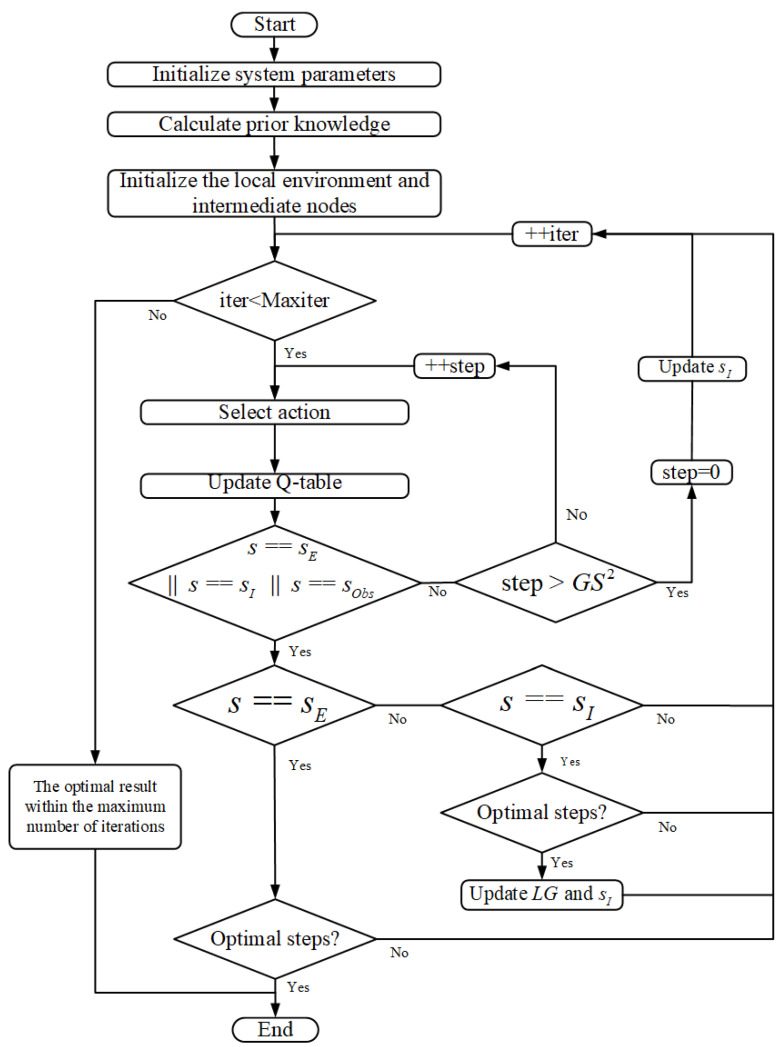
The flow of the CLSQL algorithm.

Figure 5 describes the flow of the CLSQL algorithm. The mobile robot starts to search $s_{I}$ from LG containing the starting point. When it moves to $s_{I}$, it updates the new LG and $s_{I}$ and repeats the above steps until the agent reaches the destination point, which represents the end of the whole learning process. In the local environment search process, when the number of learning steps at this stage is far greater than the size of the local environment too many times or the number of iterations is excessive, that is, the optimal solution cannot be obtained, the present $s_{I}$ in the local environment is deleted, and then another new $s_{I}$ is determined again. Global optimization can be achieved only when local environment optimization is achieved. The condition of a local optimum is whether the optimal steps correspond to the diagonal distance between two points $p_{1}$ and $p_{2}$, where the diagonal distance, namely the optimal step, is defined as:

(10) $$D=\left|x_{2}-x_{1}\right|+\left|y_{2}-y_{1}\right|+\left(\sqrt{2}-2\right)\times \min\left(\left|x_{2}-x_{1}\right|,\left|y_{2}-y_{1}\right|\right).$$

## 5. Experimental Results and Performance Analysis

This section gives the simulation results of the CLSQL algorithm (implemented using Python) and verifies the validity of the algorithm. All simulation experiments of the CLSQL algorithm were performed on a Windows 10 with NVIDIA RTX 1660 GPU and 16G RAM.

In this paper, three experiments are designed to verify the feasibility of the proposed algorithm from different directions. First, CLSQL feasibility is tested via four designed environments (ENV1 to ENV4) with different arrangements of static obstacles by two type LS. Second, the CLSQL algorithm is compared with the algorithm in literature [23] on its map, and the path length and operation time were tested and compared. Third, two kinds of ablation experiments are designed. One is the convergence rate was tested in different environments and compared with other RL algorithms, which is mainly reflected in the application of CLSQL prior knowledge to other RL algorithms; another is the experimental data obtained by the CLSQL algorithm in the same environment with different local environmental parameters are discussed.

In terms of the data improvement percentage, the final results are obtained by comparing the optimal data of the proposed algorithm with the optimal data of other algorithms. The specific operation of the experiment is as follows:

- Experiment 1 was conducted in four random static environments to verify the feasibility of CLSQL. It proves that different LS can be effectively applied in different environments and obtain satisfactory results.
- Experiment 2 is based on the map proposed in the literature [23] for comparison. In this paper, we adopt a grid map for modeling, so rasterization of the map is required. Note that the approach presented in [23] is evaluated on a PC using MATLAB R2014a software for AMD A10 Quad Core with 2.5 GHz. Six different maps T1-T6 were tested, including the CLSQL algorithm, the Improved Q-learning with flower pollination algorithm (IQ-FPA), QL, and the Improved Decentralized QL (IDQ), to verify and compare the performance of different algorithms in terms of path length and computation time. This experiment can clearly show the advantages of the proposed algorithm in path planning and computation time.
- Experiment 3 builds two kinds of environment maps and focuses on verifying the improvement in search efficiency and iteration times between the CLSQL algorithm and the QL-based algorithm in the obstacle-free or obstacle maps of different specifications. The experiment further proves that the proposed algorithm can achieve a better path effect and iteration efficiency for environment maps of any complexity. The other one, which sets different local environmental parameters, uses the CLSQL algorithm to conduct an ablation experiment in the same environment and sums up the effect of the proposed algorithm relevant to different local environmental parameters.

### 5.1. Feasibility Experiment

**Figure 6:**
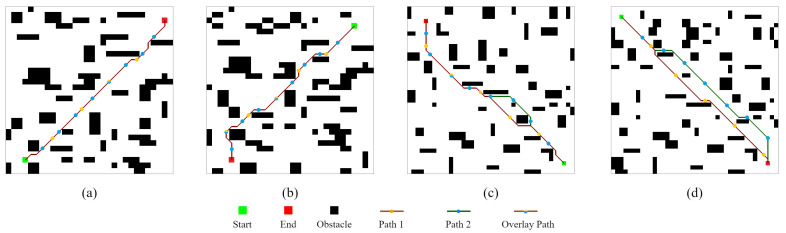
Obtained paths by the proposed CLSQL. (**a**–**d**) are the obtained path in Env1 to Env4.

Figure 6 shows the preliminary results of feasibility testing of the proposed CLSQL algorithm in different environments. The size of Env1 and Env2 is 30×30, Env3 and Env4 is 40×40, and the obstacles from Env1 to Env4 are random and chaotic. The planned path obviously achieved the effect of obstacle avoidance and satisfactory results. Meanwhile, the average computation time for all cases are 0.39 s, 0.41 s, 0.59 s, 0.37 s, and 0.56 s, respectively. Thus, with the proposed CLSQL, the mobile robot can quickly search and obtain the planned path. Moreover, CLSQL produces different or the same planned path according to different LS; it does not affect the mobile robot’s move from the starting point to the destination point, which is indisputable. As shown in Figure 6a,b, although the planned path is the same, there are different intermediate nodes, which will have different search efficiency problems, and we will discuss them in the following chapters.

Table 1 shows results on environments Env1 to Env4. The performance of CLSQL is represented by the average of 20 independent runs. As show in Table 1, it is obviously seen that the planned path can be obtained quickly under any LS condition; the length difference between the planned paths is not large. It demonstrates that CLSQL has high stability.

### 5.2. Effectiveness Experiment

**Figure 7:**
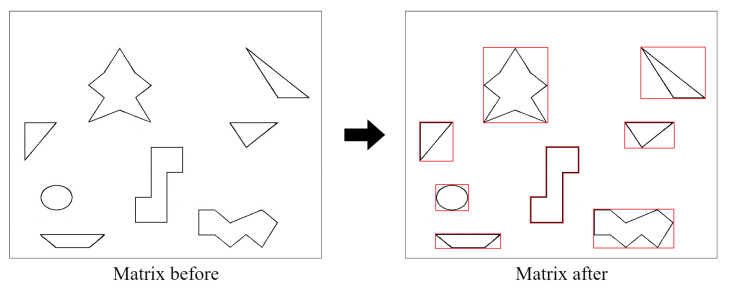
Rasterization of the map.

The environmental map introduced in the article [23] was used and the CLSQL algorithm was compared. Theoretically, the relevant same starting and destination points are used in all environmental maps. There are several obstacles in this set of environmental maps that are irregular. Therefore, to test the proposed algorithm, all irregular obstacles are rectangular, as shown in Figure 7.

**Figure 8:**
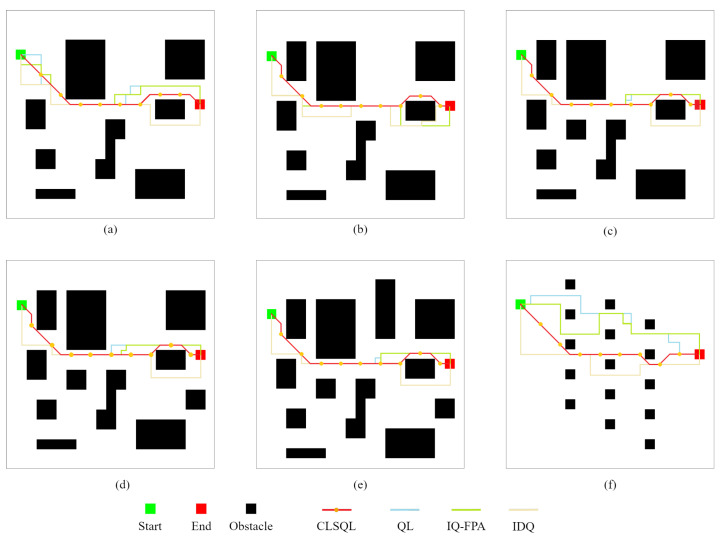
Experimental comparison of path results. (**a**–**f**) are the obtained path by different algorithms in T1-T6.

Figure 8 shows the 20 × 20 size map T1-T6 with 8, 9, 10, 11, 12, and 15 obstacles and the planned path results of different algorithms. In all of the map cases above, the mobile robot has the same starting point and destination point. Table 2 summarizes the performance comparison of the CLSQL algorithm, IQ-FPA [23], QL [15], and IDQ [35]. Table 2 also illustrates the results of 30 independent experiments using the IQ-FPA algorithm. It should be noted that the path length and operation time bits of the IQ-FPA algorithm are the average values of 30 independent experiments.

**Figure 9:**
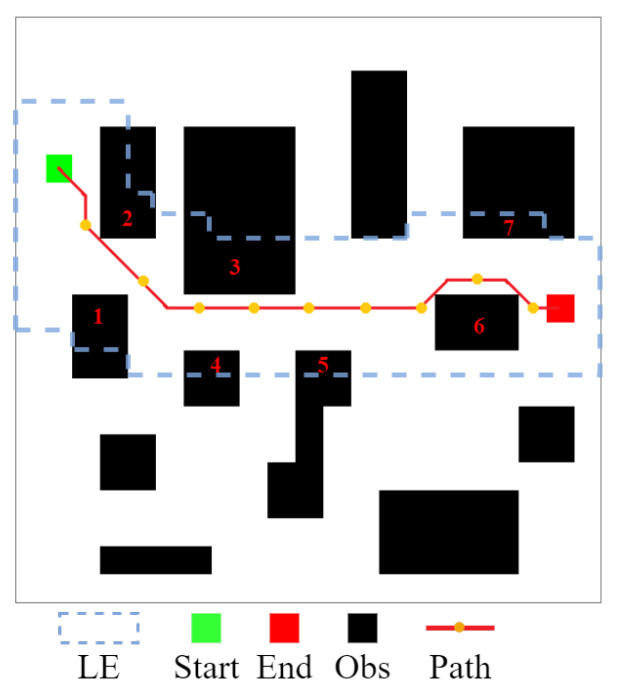
Obstacle information of the CLSQL algorithm.

By looking at the calculation time shown in Table 2, when using IDQ and IQ-FPA, the calculation time increases as obstacles increase, while the QL performance trend is reversed. In addition, the common characteristic of the above three algorithms is that their calculation time is relatively high in all map environments, while the CLSQL algorithm can quickly complete path search in all map environments. Although the hardware performance in this paper surpasses that in the literature [23], the calculation time of the CLSQL algorithm is improved by 88.50% to 91.75%, which is not only due to the improvement in hardware performance. This is because the local search policy adopted by the proposed algorithm greatly reduces the useless search space and guides the agent to learn effectively from the target according to prior knowledge. For example, in the CLSQL algorithm with the local search policy, only some obstacles of information will be added to the relevant local environment. As shown in Figure 9, only the information of 7 obstacles is searched in the T5 with the most obstacles, and the blue dashed line is composed of multiple local environments. However, the other three algorithms will take all obstacle information into consideration in the search process in the worst case. Therefore, by using the CLSQL algorithm based on a continuous local search policy, mobile robots can conduct the purposeful search in a short computation time to find the optimal path.

At the same time, it can be observed in Table 2 that the proposed optimization algorithm also shows satisfactory results in terms of path length. This is because the CLSQL algorithm adopts 8 moving directions, compared with 4 moving directions. In objective circumstances, this will increase the dimension of the algorithm, but because of the local search policy, it can comprehensively reduce the dimension required by the algorithm. As seen intuitively from Figure 8, there are many tortuous paths in the path results obtained by the QL, IQ-FPA, and IDQ algorithms, while the proposed algorithm does not yield them. In Figure 8b–e, the CLSQL algorithm obtained the same path length; although the number of obstacles was increased based on the same map environment, the result was not affected. In contrast, other algorithms are negatively affected by additional obstacles (new obstacles), which increase the calculation time and path length of the algorithm to varying degrees.

The simulation results obtained in Experiment 1 show that (i) the CLSQL algorithm can quickly generate optimal or approximate optimal paths. (ii) The path planning result of the CLSQL algorithm is relatively stable, which can reduce the influence of noncritical obstacles (obstacles far away from the path) and improve search efficiency. (iii) The prior knowledge, local search policy, and improved dynamically adjusting ε-greedy policy adopted by the proposed algorithm both provide strong support for path planning results. (iv) Compared with the latest work, the CLSQL algorithm is significantly improved in terms of both path length and computation time.

### 5.3. Ablation Experiment

**Figure 10:**
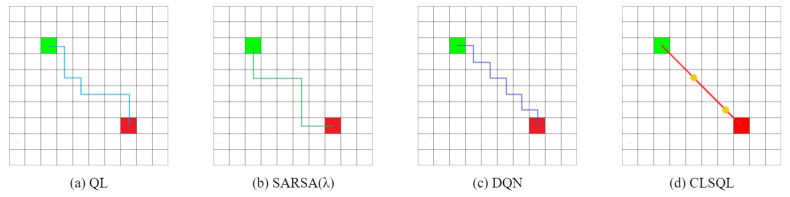
The optimal path of B1.

**Figure 11:**
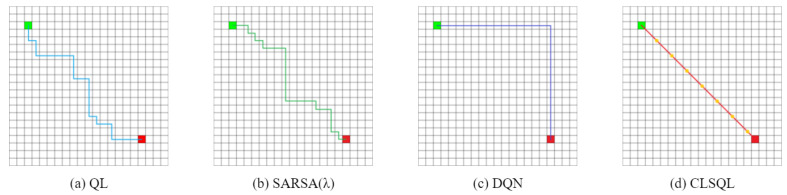
The optimal path of B2.

TheObstacle-freeMap:Figure 10 and Figure 11 show the two obstacle-free maps B1 and B2 and the optimal path obtained by using QL [15], SARSA(λ) [29], DQN [22], and CLSQL algorithm, where the green square represents the starting point, the red square represents the destination point, and the yellow circle represents the intermediate point. Paths with different colors represent the results of different algorithms. Since B1 and B2 are obstacle-free maps, grid lines are added to enhance the visualization of the maps. Table 3 and Table 4 record the average data and standard deviation of the four algorithms, respectively. Intuitively, as the map environment increases, it takes longer to compute.

Both Figure 10 and Figure 11 are optimal path graphs obtained by the four algorithms after several independent experiments. Due to the uncertainty of the results generated by reinforcement learning each time, the optimal results are selected to display the above-visualized results, but this does not affect the reliability of the experimental data.

It can be seen from Figure 10 that the CLSQL algorithm can plan the simplest and most direct path, while the paths generated by other algorithms all have turns of varying degrees, as does Figure 11. Figure 10d and Figure 11d show that the CLSQL algorithm can determine the shortest path from the starting point and destination point of the figure because the proposed algorithm at the time of Q-table initialization increases the relevant prior knowledge and increases the purpose of the search process so that in each stage, it can make the mobile robot constantly move toward the direction of the destination point. Remarkably, the DQN algorithm in Figure 11c also obtains a relatively simple path, because the neural network parameters of the DQN algorithm are updated after several learning iterations, which results in its selection policy remaining unchanged for some time, thus suddenly achieving a good path effect in certain learning situations.

**Figure 12:**
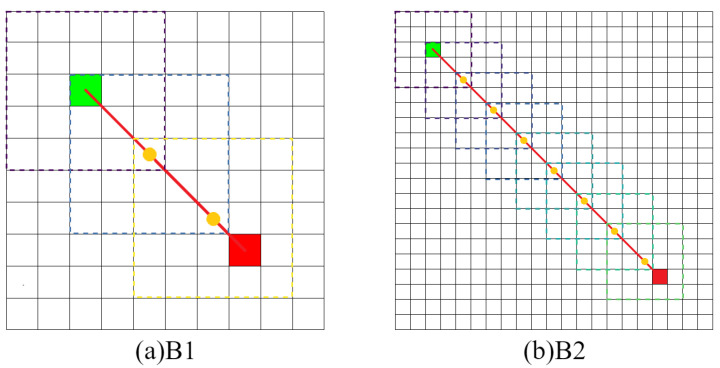
Local environment information of obstacle-free map.

Table 3 shows that in the obstacle-free map, the proposed algorithm is superior to other algorithms to varying degrees in the above four kinds of performance. In terms of the average path length, both QL and SARSA(λ) in B2 show that the optimal path result cannot be planned within the maximum number of iterations, resulting in a high average path length. The CLSQL algorithm shows satisfactory results in B1 and B2. In terms of the average calculation time, the CLSQL algorithm only requires 0.09 s and 0.26 s to quickly complete the convergence in B1 and B2, respectively, but increases by 35.71% and 33.33%, respectively. This is because of the relationship between map size and complexity, so QL and SARSA(λ) perform well, thus limiting the upper limit of the CLSQL algorithm improvement. DQN involves neural network learning, so the computation time does not exhibit strong performance. In terms of average iteration, the CLSQL algorithm improved by more than 60%, and in terms of average total steps, it improved by more than 99%. This is because the other three algorithms can only conduct a blind search in the map with no valid information, leading to duplication and invalid information in the preliminary search process. The CLSQL algorithm initializes Q-values based on prior knowledge to strengthen the purpose of the search. Second, the search space is greatly reduced by searching the present intermediate point in the local environment until the destination point is reached through several local environments and intermediate points. For example, in Figure 10d, the proposed algorithm adopts the local search policy to learn and only searches 57% of the map information in the environment at most, while the other three algorithms search all the map information at most. Similarly, in Figure 11d, the proposed algorithm searches only 34.25% of the map information in the environment at most. Figure 12 shows the local environment that may be searched in the local search process of the CLSQL algorithm. The color matrix is the local environment involved in each stage of the proposed algorithm. The lighter the color is, the closer the mobile robot is to the destination point.

In Table 4, the premise that the standard deviation of the path length of the QL, SARSA(λ), and DQN algorithms is 0 or very small is obtained based on a large number of iterations, which is not worthwhile. It can also be seen that in many different experiments, the standard deviation of the proposed algorithm can show that its overall performance exhibits good stability.

**Figure 13:**
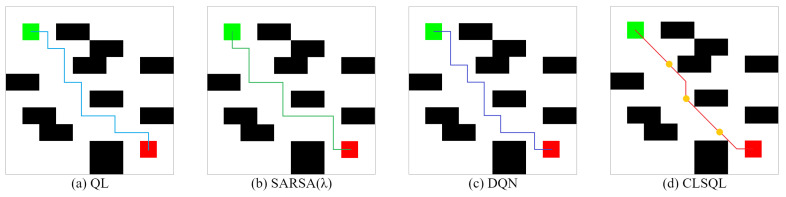
The optimal path of M1.

**Figure 14:**
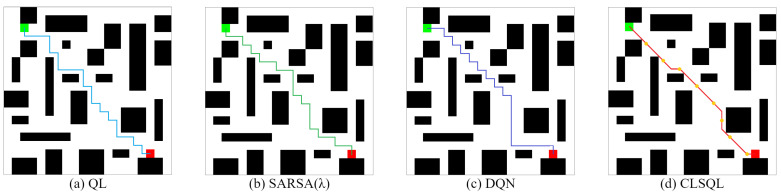
The optimal path of M2.

TheObstacleMap:Figure 13 and Figure 14 show two obstacle maps M1 (8 obstacles) and M2 (24 obstacles) and the optimal path obtained by using QL, SARSA(λ), DQN, and the CLSQL algorithm. It is worth noting that in maps M1 and M2, QL and SARSA (λ) algorithms are added with the same prior knowledge as the CLSQL algorithm to ensure that the local search efficiency of the proposed algorithm is verified under the same conditions.

It can be seen from Figure 13 and Figure 14 that the path results of the four algorithms in the two obstacle maps are tortuous. This is because the complexity of M1 and M2 is high and there are many narrow roads in the environment itself, so the algorithm has to obtain the optimal path from these narrow and complex roads.

As seen from Table 5, the CLSQL algorithm still maintains absolute advantages over other algorithms in terms of average path length and average calculation time. After adding a prior knowledge to the QL and SARSA (λ), the average path length and average computing time maintain good performance, and the average number of iterations and average total steps are greatly reduced. The CLSQL algorithm is mostly inferior to the QL algorithm in terms of the average iterations and the average total steps. This is because the CLSQL algorithm is an iterative search conducted in the continuous local environment, iterative efficiency depends on the size of the local environment and the position of the intermediate points, but in the larger, more complex M2, the CLSQL algorithm can improve the average total steps by 68.49%. At the same time, the DQN algorithm without prior knowledge to initialize the Q-table still maintains a large average number of iterations and average total steps.

In Table 6, the reason why the standard deviation of the path length of the QL, SARSA(λ), and DQN algorithms are 0 or very small is the same as in the obstacle-free map. The stability of the average iterations and average total steps of the QL and SARSA(λ) algorithms is greatly improved with the support of prior knowledge. At the same time, in terms of the aspect of standard deviation, it is further demonstrated that the CLSQL algorithm can perform more stable than other algorithms in any specification map, which verifies its advantage that it is not affected by the size and complexity of the environment (number of obstacles).

**Figure 15:**
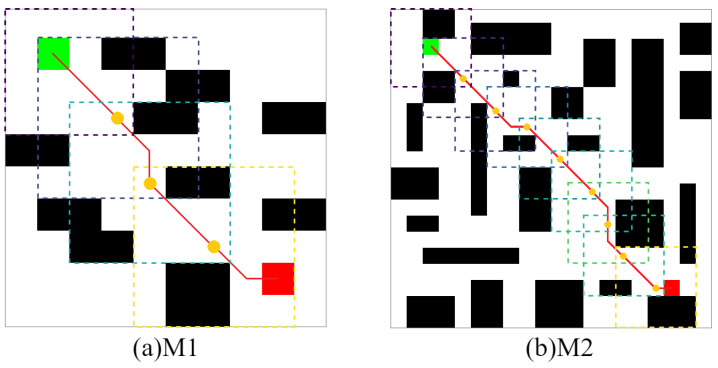
Local environment information of obstacle maps.

Figure 15 shows that the CLSQL algorithm only searched 61% and 36% of the map environment in M1 and M2, respectively. Although the size of the search environment was reduced, compared with the QL and SARSA(λ) algorithms which added prior knowledge, the average number of iterations and average total steps of the proposed algorithm did not achieve ideal results because (i) in a complex environment with small size, the effect of prior knowledge is more direct and effective than the search efficiency of the local environment. (ii) The LS adopted by the CLSQL algorithm in the first Ablation Experiment is 5, which is not the most appropriate LS, to maintain the relative stability of the experiment. The second Ablation Experiment proves that different LS will produce different experimental results. At the same time, the reason why the average total step number of the CLSQL algorithm is larger in M1 than in M2 is that the appropriate LS is not adopted, thus increasing the search space a small amount and reducing the search efficiency.

Therefore, some interesting results and inspirations can be obtained from the chart of obstacle-free maps, such as (i) the CLSQL can achieve optimal path planning results within a short time for maps of any size and complexity; (ii) prior knowledge in the environment offers strong support for improving search efficiency; (iii) with the gradual increase in environmental complexity and environment size, the local search policy for the CLSQL algorithm will be more likely to play a positive role; and (iv) only when appropriate local environment size and intermediate nodes are selected can the optimal path and performance be obtained by the proposed algorithm.

TheSameEnvironmentMap: In the second Ablation Experiment, the CLSQL algorithm was used to test the LS of different sizes of two self-designed maps with obstacles, Map1(28 obstacles) and Map2(39 obstacles), to verify that different LS have different experimental effects in the same environment. The following experimental data are the average data obtained after 10 independent experiments. The size of Map1 is 30 × 30, and Map2 is 40 × 40.

**Figure 16:**
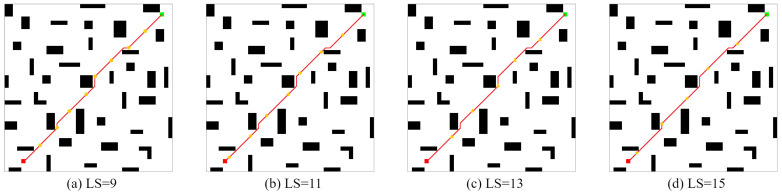
The optimal path of Map1 by the CLSQL.

**Figure 17:**
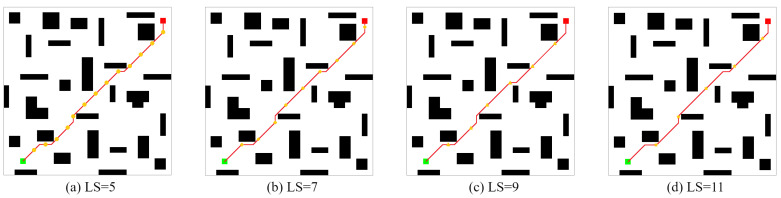
The optimal path of Map2 by the CLSQL.

It can be seen from Figure 16 and Figure 17 that in Map1 and Map2, the final path results are consistent when adopting local environments with different sizes because each iteration process in local environments with different sizes follows the prior knowledge of the same environment.

It can be concluded from Table 7 that the average path length of the CLSQL algorithm has strong consistency in local environments like Map1 and Map2 with different sizes. In Map2, the slightly higher average path length in LS = 9 and LS = 11 is due to the occurrence of nonoptimal results in some experiments, resulting in a slightly greater path length than the average. With the increase in map and environment complexity, the larger the local environment is, the shorter the average calculation time of the CLSQL algorithm. This is because in an environment of the same size, the larger the size of the local environment is, the smaller the number of local environments that exist, thus reducing the calculation time, and vice versa. At the same time, in a larger local environment, it is easy to complete path optimization in fewer average iterations. In contrast, in the two maps, when the algorithm adopts the local environment with the smallest size, the algorithm uses the least average total steps to complete the path-finding work. In Map1, when LS = 7 and LS = 9, the average number of iterations and the average total number of steps are abnormally large because, under this premise, the CLSQL algorithm cannot immediately find the optimal intermediate point, resulting in a large number of repeated searches in some local environments, which reduces the search efficiency. However, in Map2, there is little difference between the sizes of the four LS, so the difference between the average number of iterations and the average total number of steps is within a reasonable range.

In terms of Table 7 and Table 8, the larger the local environment is, the more likely it is to yield better experimental data and, conversely, the more stable the experimental data. To summarize, the comparative experimental results proved that the most suitable local environment is not necessarily maximal or minimal. To produce optimal results, it is necessary to first consider the environment complexity of different maps; there will always be a local environment suitable for the size of the present environment, and finally, a suitable path planning result will be obtained.

To summarize from the obtained simulation results, (i) the proposed CLSQL is able to generate the planned path quickly, (ii) the CLSQL method is independent, that is, the previously planned path is not affected by the subsequent search, which is helpful to the search efficiency of the algorithm, and finally, (iii) the proposed CLSQL performance is significantly improved compared to RL-based work, including path length and algorithm efficiency.

## 6. Conclusions

In this paper, we efficiently solved the path planning problem of mobile robots in a static environment and proposed an improved Q-learning method based on a continuous local search policy for mobile robots to quickly generate optimal paths in static obstacle maps, especially in a complex environment. Simulation results show that (i) by comparison with the relevant QL algorithm, the CLSQL algorithm achieves good results in terms of path length, calculation time, iteration number, and total step number. (ii) By integrating the prior knowledge of the environment into the QL algorithm, the initialized Q-table plays a crucial role in the environment search, as well as using the optimized dynamically adjusting ε-greedy policy. This can speed up the mobile robot’s search process. At the same time, in a larger complex environment, local search policy will play an increasingly important role. (iii) For environmental maps of different sizes and complexities, there will always be a relatively appropriate local environment to adapt to the present environment. In summary, the CLSQL algorithm can quickly provide the planned path in maps of different size and complexity. From this paper, it can be concluded that the possible future direction is to strengthen the adaptive capacity of the local environment fitness, especially when there are many complex factors in the environment. The local environment can be arbitrarily changed in size in the search process to adapt to a different environment.

## Figures and Tables

**Table 1:** Performances of CLSQL on the environments Env1 to Env4.

Environment	LS (Unit)	Path Length (Unit)	Computation Time(s)
Env1	7	36.53	0.41
	11	36.53	0.38
Env2	7	36.87	0.43
	11	36.87	0.39
Env3	11	53.11	0.60
	15	52.47	0.59
Env4	11	51.14	0.58
	15	50.67	0.54

**Table 2:** Performance Comparison of Map T1–T6.

Environment	Average Path Length (Unit)					Average Computation Time (s)				
	QL	IDQ	IQ-FPA	CLSQL	Improvement Percentage(%)	QL	IDQ	IQ-FPA	CLSQL	Improvement Percentage(%)
T1(8 obstacles)	28.93	26.27	29.33	20.90	20.44	4.06	9.26	3.52	0.30	91.48
T2(9 obstacles)	30.67	26.37	31.27	21.49	18.51	4.04	10.05	3.62	0.31	91.44
T3(10 obstacles)	30.00	26.00	29.60	21.49	17.35	3.89	10.28	3.88	0.32	91.75
T4(11 obstacles)	27.67	26.27	28.73	21.49	18.20	3.64	27.10	3.98	0.32	91.21
T5(12 obstacles)	27.67	26.53	29.00	21.49	19.00	3.71	23.39	4.00	0.31	91.64
T6(15 obstacles)	30.07	26.00	26.80	21.04	19.08	3.23	2.87	3.66	0.33	88.50

**Table 3:** Average Performance Comparison of Obstacle-free Maps.

Environment	Average Path Length (Unit)		Average Computation Time (s)		Average Iteration(Unit)		Average Total Step (Unit)	
	B1	B2	B1	B2	B1	B2	B1	B2
QL	10	31.4	0.14	0.39	20.4	114.5	3592.0	52451.8
SARSA(λ)	10	36.4	0.15	0.52	88.8	525.2	4699.0	28570.1
DQN	10	30	0.49	1.19	9.4	24.5	9058.0	15363.3
CLSQL	7.07	21.21	0.09	0.26	3.3	9.4	8.38	27.54
Improvement (%)	29.30	29.30	35.71	33.33	64.89	61.63	99.77	99.82

**Table 4:** Standard Deviation Performance Comparison of Obstacle-free Maps.

Environment	Path Length (Unit)		Computation Time (s)		Iteration (Unit)		Total Step (Unit)	
	B1	B2	B1	B2	B1	B2	B1	B2
QL	0.00	2.01	0.01	0.12	5.39	16.24	720.05	8783.63
SARSA(λ)	0.00	4.45	0.02	0.06	65.05	397.83	1277.49	19974.05
DQN	0.00	0.00	0.15	0.03	4.25	18.90	2063.27	9622.40
CLSQL	0.00	0.00	0.01	0.02	0.46	0.92	2.03	4.73

**Table 5:** Average Performance Comparison of Obstacle Maps.

Environment	Average Path Length (Unit)		Average Computation Time (s)		Average Iteration (Unit)		Average Total Step (Unit)	
	M1	M2	M1	M2	M1	M2	M1	M2
QL	14	30	0.19	0.41	3.3	7.8	31.3	117
SARSA(λ)	14	30	0.21	0.43	6.7	13.6	71.1	254.6
DQN	14	30.4	0.55	1.22	313.2	634.2	4149.9	8211
CLSQL	10.49	22.38	0.14	0.28	12.7	14.5	42.85	36.87
Improvement (%)	25.07	25.40	26.32	31.71	−284.85	−85.90	−36.90	68.49

**Table 6:** Standard Deviation Performance Comparison of Obstacle Maps.

Environment	Path Length (Unit)		Computation Time (s)		Iteration (Unit)		Total Step (Unit)	
	M1	M2	M1	M2	M1	M2	M1	M2
QL	0	0	0.01	0.02	0.80	1.17	9.06	13.95
SARSA(λ)	0	0	0.01	0.02	1.95	4.03	29.73	102.44
DQN	0	1.20	0.04	0.06	154.50	160.01	2322.13	5409.54
CLSQL	0	0	0.01	0.01	1.42	1.20	5.48	3.27

**Table 7:** Local Environment Average Performance Comparison.

Environment	LS (Unit)	Average Path Length (Unit)	Average Computation Time (s)	Average Iteration (Unit)	Average Total Step (Unit)
Map1	5	37.11	0.47	47.1	162.52
	7	37.11	0.51	71.5	415.79
	9	37.11	0.47	57.2	362.83
	11	37.11	0.42	36.1	258.32
Map2	9	49.31	0.56	39.3	308.50
	11	49.25	0.53	40.4	370.79
	13	49.31	0.52	35.9	389.57
	15	49.25	0.52	38.9	445.44

**Table 8:** Local Environment Average Performance Standard Deviation Comparison.

Environment	LS (Unit)	Average Path Length (Unit)	Average Computation Time (s)	Average Iteration (Unit)	Average Total Step (Unit)
Map1	5	0	1.30	2.39	9.23
	7	0	1.86	3.75	32.31
	9	0	2.45	4.77	28.60
	11	0	3.04	4.81	32.32
Map2	9	0.17	2.43	7.11	75.40
	11	0	3.01	5.14	95.32
	13	0.17	3.59	14.66	192.21
	15	0	4.16	9.07	140.80

## Data Availability

Not applicable.
